# Effect of dietary *Clostridium butyricum* supplementation on growth performance, immune function, and intestinal health of hybrid grouper (*Epinephelus fuscoguttatus* ♀ × *Epinephelus lanceolatus* ♂)

**DOI:** 10.3389/fimmu.2025.1557256

**Published:** 2025-02-26

**Authors:** Weijun Chen, Tao Song, Dong Li, Mingfan Chen, Pan Wang, Jidan Ye

**Affiliations:** Fisheries College of Jimei University, Xiamen Key Laboratory for Feed Quality Testing and Safety Evaluation, Xiamen, China

**Keywords:** *Clostridium butyricum*, hybrid grouper, growth, immune function, gut microbiota

## Abstract

**Introduction:**

The aim of this study is to investigate the effects of supplementing *Clostridium butyricum* (*C. butyricum*) on hybrid grouper (*Epinephelus fuscoguttatus* ♀ × *Epinephelus lanceolatus* ♂), with a particular focus on its impact on growth performance, blood composition, intestinal antioxidant capacity, gut microbiota, tight junction protein (ZO-1) expression, and inflammatory gene expression. The study seeks to uncover the potential health benefits of C. butyricum supplementation for hybrid grouper.

**Methods:**

The experiment included four groups: a control group (CON) and three experimental groups, each supplemented with different strains of *C. butyricum* (KM, DZN, and CLH), with a concentration of 1 × 10^⁷^ colony-forming units per gram. These groups were designated as CB1 (KM), CB2 (DZN), and CB3 (CLH). The study evaluated growth performance, blood composition, intestinal antioxidant capacity, gut microbiota, ZO-1 protein expression, and inflammatory gene expression (*IL-1β* and *Ikk-β*).

**Result:**

The results indicated that supplementation with *C. butyricum* had no significant effect on body weight gain (WG), feed efficiency (FE), or body composition. However, the CB3 group significantly increased the activity of superoxide dismutase (SOD) and glutathione peroxidase (GSH-Px) in the intestine, as well as the expression of ZO-1. In addition, the CB3 group significantly increased serum lysozyme (LZM) activity, complement 4 (C4) levels, and immunoglobulin M (IgM) concentration, while significantly reducing the expression of pro-inflammatory genes (*IL-1β* and *Ikk-β*). After supplementation with *C. butyricum*, the level of malondialdehyde (MDA) in the intestine was significantly lower than that in the control group, indicating a reduction in intestinal oxidative stress. Supplementation with *C. butyricum* also altered the composition of the gut microbiota, promoting the growth of beneficial bacteria and inhibiting pathogenic bacteria, thereby further enhancing ZO-1 expression and intestinal barrier function.

**Discussion:**

This study suggests that supplementing *C. butyricum* has a significant immunomodulatory effect on hybrid grouper, enhancing serum immune parameters, alleviating intestinal inflammation and oxidative stress, and promoting intestinal health. Although no significant impact was observed on growth performance, the role of *C. butyricum* in improving intestinal barrier function and modulating the gut microbiota highlights its potential for enhancing fish health.

## 1 Introduction

It is well known that gut is an important organ of fish that performs the absorption and metabolism of nutrients and the essential maintenance of gut health and homeostasis of fish in the healthy state (1) as a matter of gut microbiota contributions (2, 3). For instance, gut microbiota participate in the development and repair of the host’s gut epithelia and maintain the integrity of the gut barrier (4), resisting the invasion of pathogenic bacteria via inhibiting the proliferation of pathogenic bacteria (5–7). In contrast, unhealthy gut is linked to the imbalance of gut flora, accompanied with disruption of gut mucosal barrier function and the colonization and proliferation of pathogenic microbiota, eventually leading to diarrhea or enteritis (8). The practice of intensive farming has become an important factor contributing to the unhealthy conditions of farmed animals, resulting in the development of many diseases (9). Therefore, how to manipulate gut microbiota to achieve a more favorable balance received widespread attention, from a perspective view of improving health by feed measures (10).

Historically, antibiotics have been used extensively for routine disease prevention and control (11). However, there is still an urgent shift to use environmentally friendly and safe measures to replace antibiotics. This is not only a drug resistance issue, but also a major concern for human health (12, 13). Probiotics are live bacteria that confer a health benefit on animals as their consumption is sufficient (14). As a functional substance, they can regulate gut microbiota by lowering gut pH, providing nutrients, secreting antibacterial substances, adhering to pathogenic bacteria and regulating host immune responses (15, 16). Numerous feeding experiments have shown that the addition of probiotics to feed can improve growth performance and health status of fish and shellfish, enhancing resistance to pathogen infections (17–24). With these potential health benefits of probiotics on aquatic animals, the use of probiotics in aquaculture has gained increasing attention in recent years.

*Clostridium butyricum* (*C. butyricum*) is a specific anaerobic, Gram-positive, which is widely present in animals and soil (25, 26). It not only has excellent environmental adaptability to heat, acidity, alkalinity, and bile salts, but can also regulate intestinal function by secreting bioactive substances such as short chain fatty acids, B vitamins, digestive enzymes, etc. in the intestine (27). It is precisely because of many excellent characteristics mentioned above that *C. butyricum* has attracted widespread attention from aquaculture as a highly promising feed probiotic (11, 25, 28–35). At present, many studies have emphasized the positive effects of adding *C. butyricum* to feed, particularly on aquatic animals (9, 29). However, limited direct comparative studies have been conducted the effects of *C. butyricum* strains derived from different sources. The different origins of *C. butyricum* strains may also have varying effects on fish growth and intestinal health. Over the past three decades, probiotic microorganisms have been widely used in aquaculture, sourced from both host and non-host-derived organisms (36, 37). Host-derived probiotics have demonstrated significant potential for application in feed due to their unique biochemical characteristics (36). When selecting suitable probiotics, various factors should be evaluated, including microbial type, source, strain type, biological activity, and spore formation, all of which are crucial for their successful application in aquaculture (38, 39). Therefore, selecting the appropriate *C. butyricum* strains remains a critical step for its effective application in aquaculture.

The hybrid grouper (*E. lanceolatus ♂ × E. fuscoguttatus ♀*) is a predatory marine fish that is extensively cultivated in China and other Southeast Asian coastal nations due to its good flavor and benefits for intensive farming (40). However, the frequency of disease occurrence is increasing with the continuous promotion of intensive farming of hybrid grouper, which brings about difficulties in disease prevention and control, and has become one of the major issues that need to be addressed in the healthy culture of grouper. In particular, three types of enteric pathogen such as *Vibrio anguillarum*, *Edwardsiella tarda* and *Aeromonas hydrophila* pose a major threat to grouper enteritis (41). Recent studies reported that *C. butyricum* addition in feed can improve growth performance and intestinal health in hybrid grouper fed with commercial feed or the feed with fish meal replacement by cottonseed protein concentrate via promoting digestive ability, gut morphometry, and disease resistance (25, 42). However, it remains unknown how *C. butyricum* exerts the beneficial effects on growth and gut health improvement in this species. Therefore, with the use of microbiome, this study explored the mechanisms by which *C. butyricum* improves growth and intestinal health of hybrid grouper. The present study will provide a scientific basis for the rational application of *C. butyricum* in the diet of hybrid grouper.

## 2 Materials and methods

### 2.1 Experimental diets

Three distinct strains of *C. butyricum* were purchased as powdered products for this experiment. The KM strain (*C. butyricum* strain number GDMCC NO 1.4721, with a viable bacterial concentration of 1 × 10⁹ cfu/g) was isolated from the intestinal tract of fish in natural aquatic environments and obtained from Guangzhou Kemu Biotechnology Co., Ltd. The DZN strain (*C. butyricum* strain number GDMCC NO M2016421, with a viable bacterial concentration of 1 × 10⁹ cfu/g) was sourced from Guangdong Dazhe Agricultural Biotechnology Co., Ltd., and isolated from the intestines of healthy terrestrial animals. The CLH strain (*C. butyricum* strain number GDMCC NO 1.676, with a viable bacterial concentration of 1 × 10⁹ cfu/g) was isolated from natural aquatic environments and obtained from Zhanjiang Hengxing Aquaculture Technology Service Co., Ltd. The control diet (CON) was formulated using fish meal and gelatin as the protein sources and fish oil, soybean oil, and soybean lecithin as the fat sources (**Table 1**). Based on this formulation, three strains of *C. butyricum* (KM (CB1), DZN (CB2), and CLH (CB3), respectively) were evenly sprayed onto the diets to achieve a viable bacterial count of 1 × 10^7^ cfu/g. The feed ingredients were ground using a ZFJ-300 grinder (Jiangyin Ruizong Machinery Manufacturing Co., Ltd., Jiangyin, Jiangsu, China) passed through a 60-mesh sieve, weighed, and homogenized. Liquid ingredients were then added to the dry feed ingredients to prepare malt syrup. The dough was shaped into strips using a double-helix feed extruder (F-76, Guangzhou Huagong Optoelectronic Technology Co., Ltd., Guangzhou, Guangdong, China) and a feed pellet-forming machine (GY-500, Changzhou Beicheng Drying Equipment Engineering Co., Ltd., Changzhou, Jiangsu, China), and then pelletized using 2.5 mm and 5 mm dies. The feed was dried in a ventilated oven at 55°C for 24 hours to reduce its moisture content to below 100 g/kg. The feed was then ventilated at room temperature for 24 hours, sealed in plastic bags, and stored in a refrigerator at -20°C.

**Table 1:** Composition of the basal diet (as-dry basis, %).

Ingredients	Composition
Fish meal	52.00
Casein	11.98
Gelatin	3.00
Corn starch	17.73
Soybean oil	3.50
Fish oil	0.82
Soybean lecithin	2.00
Ca(H_2_PO_4_)_2_	1.50
Choline chloride	0.40
Vitamin C	0.02
Vitamin mix	0.40
Mineral mix	0.50
Microcrystalline cellulose	6.15
Nutrient level	
Crude protein	49.90
Crude lipid	13.36
Ash	11.63

Fish oil, soybean oil, corn starch, soybean lecithin, fish meal (crude protein 69.01% and crude lipid 9.05%), vitamin premix, and mineral premix were purchased from Jiakang Feed Co., Ltd, Xiamen, Fujian, China; Gelatin (food grade) was obtained from Shangshui Fuyuan Gelatin Co., Ltd.; Casein (food grade) was purchased from Gansu Hualing Dairy Co., Ltd.

### 2.2 Feeding experiment

The experiment was conducted at the fishery base of Dabeinong Group, located in Zhaoan County, Zhangzhou City, Fujian Province, China. Prior to the experiment, juvenile hybrid groupers were raised in blue polypropylene tanks and fed with the control diet (CON) for two weeks. At the commencement of the experiment, 300 juvenile hybrid groupers, with an initial average weight of 24.91 ± 0.01 g, were randomly distributed into 12 tanks (500 L/tank), with a density of 25 fish per tank. Under the natural photoperiod, triplicate groups of tanks were hand-fed one of the diets to satiation twice daily, at 8:00 AM and 5:00 PM,for a duration of 56 days. Any excess feed was collected 30 minutes after each meal, dried at 65℃ for 24 hours, and then weighed to calculate the feed intake.

### 2.3 Sample collection and index determination

Upon completion of the culture experiment, the total weight of the fish in each tank was measured, and the number of fish in each tank was recorded. The fish were subsequently returned to their respective tanks and subjected to a 2-day feeding cessation to mitigate the stress effects induced by the weighing process, prior to sampling. At the time of sampling, 10 fish were randomly selected from each tank, anesthetized with eugenol and then weighed, measured, and recorded. Blood was drawn from the tail vein using a 1 mL syringe and collected in a 1.5 mL centrifuge tube. The blood allowed to stand for 12 h at 3000 rpm, centrifuged at 4°C for 10 min, and then combined with serum by tank. The sample was stored in a refrigerator at -80°C for serum biochemical index determination. The liver of each fish was then excised, weighed and recorded for the calculation of the hepatosomatic index (HSI). Intestinal tissues (proximal intestine, middle intestine and distal intestine) from the same portion of each fish were collected and stored in Bonn solution for paraffin sectioning. Intestinal tissues from four fish were collected, quickly placed in a freezing tube, frozen in liquid nitrogen, and stored in a refrigerator at -80°C. Three fish were then randomly selected and stored in a refrigerator at -20°C for determination of whole fish body composition.

Whole fish samples were processed following the method described by Ye et al. (43). Specifically, after thawing the whole fish sample, its surface was wiped with gauze, followed by weighing. The fish was then placed in a weighed aluminum box, autoclave at 121°C for 30 min, cooled, cut into pieces, dried in an oven at 65°C for 24 h, and reweighed. After 24 h at room temperature, the sample was reweighed and then crushed with a small grinder, packed in a self-sealing bag and stored in a refrigerator at -20°C. Measurements of moisture, crude protein, crude lipid and ash content in experimental diets and whole fish samples were made according to AOAC method (44).

Serum aspartate aminotransferase (AST), alanine aminotransferase (ALT), C4, IgM, and LZM and total antioxidant capacity (T-AOC), MDA, GSH-Px, catalase (CAT) and SOD in the intestine were measured using the kit from Nanjing Jiancheng Bioengineering Institute, Nanjing, Jiangsu, China according to the manufacturer’s instructions (7, 11, 18, 19, 27).

### 2.4 Intestinal histology observation

Intestinal morphology was visualized using hematoxylin and eosin (H&E) staining. Following standard fixation, intestinal tissue samples were embedded in paraffin and sectioned into 5 μm-thick slices. After dewaxing in xylene I and xylene II for 20 min each, the intestinal slices underwent gradient dehydration in ethanol baths of decreasing concentrations, with 5 min per bath. The intestinal slices were washed three times with distilled water, followed by incubation with hematoxylin for 3 min, and then rinsed again with distilled water. Gradient dehydration of the intestinal slices was also performed in ethanol baths of increasing concentrations for 3 min per bath, followed by incubation with eosin for 3 min. The slices were then transferred to xylene and immersed for 3 min to render them transparent, prior to sealing with neutral resin. Images were examined under a light microscope (Leica DM5500B), and digital photographs were captured using a digital camera (Leica DFC450) equipped with LAS AF imaging software (Version 4.3.0 Leica).

### 2.5 Intestinal microbiota analysis

Intestinal samples were collected for microbiological analysis. The E.Z.N.A.TM kit (Omega Bio-Tek, Norcross, GA, USA) was used to extract total DNA from the microbial genome of the intestinal samples. The instructions provided with the kit were followed for specific steps. A 1% agarose gel was employed to assess the concentration and purity of the extracted genomic DNA. Based on the concentration, the DNA was uniformly diluted to 15 ng/μL with sterile water. Amplification primers (338F: 5’-ACTCCTACGGGAGGCAGCAG-3’; 806R: 5’-GTGGACTACHVGGGTWTCTAAT-3’) were used to amplify total DNA using an ABI 9700 PCR instrument (Applied Biosystems, Inc., USA). An 8-bp barcode sequence was added to the 5’ end of the upstream and downstream primers to distinguish between different samples. All PCR reactions were conducted in 25 μL reaction volumes, using 12.5 μL of 2xTaq Plus Master Mix. 0.2 μM forward and reverse primers and approximately 30 ng of template DNA. The PCR reaction conditions were as follows: pre-denaturation at 94°C for 5 minutes; followed by 40 s denaturation at 94°C, 50 s of annealing at 50°C, 60 s extension at 72°C, repeated for 30 cycles, and a final extension at 72°C for 7 min. The PCR product was visualized using 1% agarose gel electrophoresis (170V 30 min). followed by automatic purification using the Agencourtam Pure XP nucleic acid purification kit (Beckman Coulter, Inc., USA). PCR libraries were constructed using the NebNext Ultra II DNA Library PrepKit (New England Biolabs, Inc., USA). The constructed library was purified using the AM Pure XP nucleic acid purification kit (Beckman Coulter, Inc., USA). The library was then sequenced on the Nextseq 2000 platform (Illumina, Inc., USA) using the PE300 sequencing strategy. The off-board data were classified according to the barcode sequence. Pear software was employed to filter and splice the sequencing data. After splicing, low-quality and chimera sequences were removed using Vsearch software, and the sequences were clustered into operational taxonomic units (OTUs) using the UPARSE algorithm, with a sequence similarity threshold of 97%. The OTU was then compared with the Silva138 database using BLAST algorithm, with an e-value threshold set to 1e-5 to obtain species classification information for each OTU. Based on the results of OTU abundance, the α diversity index and β diversity distance matrix were calculated using QIIME software, plotted with R software.

### 2.6 RNA extraction and gene expression

Total RNA was extracted from individual livers using the SYBR Premix Ex Taq Kit (Takara, Dalian, China), followed by quantification of RNA concentration and purity via spectrophotometry and quality assessment using agarose gel electrophoresis. Reverse transcription was performed with 1 μg of total RNA using a reverse transcription kit (Thermo). Target gene expression was quantified by quantitative real-time PCR (qRT-PCR) using an ABI 7500 real-time PCR Detection System (Applied Biosystems, Foster City, CA, USA) with SYBR Green Real-time PCR Master Mix (Toyobo, Shanghai, China). Primers for the amplification of gene-specific PCR products were designed using Primer-BLAST (https://www.ncbi.nlm.nih.gov/tools/primer-blast/), and primer details for qRT-PCR are provided in **Table 2**. All primers were commercially supplied by Integrated DNA Technologies (Hunan Accurate Biological Engineering Co., Ltd., Changsha, China). The real-time PCR procedure involved an initial denaturation step at 95°C for 30 s, 40 cycles at 95°C for 5 s, annealing and extension temperature at 60°C for 30 s, and the final dissociation step. The final step was performed to confirm amplification of a single product. qRT-PCR efficiency (*E*) was achieved using the equation *E*=10^(−1/slope)^-1. Gene expression results were analyzed using the 2^-ΔΔCt^ method only after primers were verified to have an efficiency of approximately 100% through amplification, with data from all treatments compared to the control group.

**Table 2:** Primers for the intestinal genes.

Genes	Forward (5’-3’)	Reverse (5’-3’)	Accession no.
*occludin*	GGAGGAGAAACAGGGAATGAACT	TCTGCTACAGCCTGGTATTTGG	KF861990.1
*claudin15a*	GGTTACATCCAAGCGTCTCG	TGCCAGCAATCCTCCCTTT	MK809394
*ZO-1*	GGCGACAGAGCAGACTTTT	CCCTGGGTTCACTCTTTGC	MK809396
*IL-1β*	ATGCCTGAGGGACTGGAACTT	CTCATCAGTCGGTGGAGTTGC	EF582837.1
*IL-10*	ATGCCTGAGGGACTGGAACTT	CTCATCAGTCGGTGGAGTTGC	EF582837.1
*NF-κB* (*P65*)	CAACGACACCACTAAGACCCAC	GTCACCAATGAGATGCGAACA	EU219847.1
*Ikk-β*	CTTTGCACCTCGTTTGTGGG	CAGCCTCAGTTTGTTGTGCC	KM669150.1
*β-actin*	GGCTACTCCTTCACCACCACA	TCTCCAAGGCAACGGGTCT	AY510710.2

### 2.7 Statistical analysis

All data were presented as mean and standard error of the mean (SEM). The data were analyzed using one-way analysis of variance (ANOVA) to assess differences between treatments, followed by the Student-Newman-Keuls multiple comparison test. Normality and homogeneity of variance were confirmed using the Kolmogorov-Smirnov and Levene’s tests, and all analyses were conducted in SPSS Statistics 25.0 (SPSS, Michigan Avenue, Chicago, IL, USA). Data expressed as percentages or ratios underwent data conversion before statistical analysis. *P*-values < 0.05 were considered statistically significant.

## 3 Results

### 3.1 Growth performance and whole-body proximate composition

**Table 3** shows that the addition of *C. butyricum* to the diet did not have no adverse effects on the growth performance and whole-body proximate composition of hybrid grouper. No significant differences were observed in WG, FE, FR, HSI, CF, SR, SGR, or whole-body proximate composition (moisture, crude protein, crude lipid and ash) among the groups (*P* > 0.05).

**Table 3:** Effects of dietary *C. butyricum* addition on growth performance and whole-body proximate composition of hybrid grouper^1^.

Parameters	Diets			
	CON	CB1	CB2	CB3
Growth performance				
IW (g/fish) ^2^	24.89 ± 0.05	24.92 ± 0.02	24.92 ± 0.02	24.90 ± 0.01
WG (%) ^2^	407.67 ± 22.65	408.13 ± 4.65	404.38 ± 2.40	405.95 ± 5.74
SGR (%/d) ^2^	2.90 ± 0.08	2.90 ± 0.16	2.89 ± 0.01	2.90 ± 0.02
FE (%) ^2^	110.82 ± 3.04	117.53 ± 4.35	118.44 ± 2.85	123.04 ± 9.49
FR (%/d) ^2^	2.15 ± 0.08	2.04 ± 0.08	2.02 ± 0.05	2.05 ± 0.04
HSI (%) ^3^	2.39 ± 0.19	2.58 ± 0.10	2.44 ± 0.31	2.37 ± 0.17
CF (g/cm^3^) ^3^	2.97 ± 0.04	2.95 ± 0.05	2.75 ± 0.01b	2.89 ± 0.07
SR(%)^3^	97.33 ± 0.05	93.33 ± 0.05	94.67 ± 0.05	96.00 ± 0.07
Whole-body proximate composition (%)				
Moisture	69.3 ± 0.12	69.85 ± 0.37	70.22 ± 0.03	69.29 ± 0.84
Crude protein	17.73 ± 0.25	17.76 ± 0.34	17.93 ± 0.13	17.80 ± 0.47
Crude lipid	8.34 ± 0.22	7.28 ± 0.02	6.82 ± 0.03	8.26 ± 0.89
Ash	4.48 ± 0.31	4.45 ± 0.22	4.52 ± 0.16	4.58 ± 0.21

^1^Statistical analysis was performed by one-way ANOVA, followed by S-N-K test.

^2^The numerical values are expressed as the means ± SEM (n = 3 tanks).

^3^The numerical values are expressed as the means ± SEM (n = 25 fish).

WG (weight gain, %) = 100 × (FW - IW)/IW.

SGR (specific growth rate, %/d)= 100 × (lnFW - lnIW)/days.

FE (feed efficiency, %) = 100 × (FW - IW)/(total feed intake).

FR (feed intake rate, %/d)=100 × (total feed intake)/[(IW+FW)/2]/days.

SR(survival rate)(%) = 100 × (final number)/(initial number).

HSI (hepatosomatic index, %) = 100 × (body length, cm/fish)/(body weight, g/fish).

CF (condition factor, g/cm^3^) = 100 ×(body weight, g/fish)/(body length, cm/fish)^3^.

IAW, initial average weight (g/fish); FAW, final average weight (g/fish).

### 3.2 Intestinal antioxidant capacity

**Table 4** demonstrates the effect of adding *C. butyricum* to the feed on the intestinal antioxidant capacity of the hybrid grouper. The activities of GSH-Px and SOD in the experimental group were significantly higher than those in the control group (*P* < 0.05). In the CON group, the CB3 group exhibited the highest values (*P* < 0.05). The addition of CB1 and CB3 to the feed significantly decreased MDA content in the intestine (*P* < 0.05), while no significant difference was observed between CB2 and CON (*P* > 0.05). No statistically significant differences were observed in the activities of CAT and T-AOC among the groups (*P* > 0.05).

**Table 4:** Effects of dietary *C. butyricum* addition on intestinal antioxidant capacity of hybrid grouper.

Parameters	Diets			
	CON	CB1	CB2	CB3
MDA (nmol/mg prot)	3.22 ± 0.73^a^	1.33 ± 0.01^b^	3.27 ± 0.91^a^	1.71 ± 0.01^b^
T-AOC (mmol/g)	1.18 ± 0.27	1.35 ± 0.08	1.13 ± 0.22	1.27 ± 0.02
CAT (U/mg prot)	21.83 ± 0.08	22.32 ± 1.80	22.37 ± 0.11	19.59 ± 0.25
SOD (U/mg prot)	26.6 ± 0.49^a^	29.5 ± 1.15^b^	35.11 ± 0.21^bc^	45.06 ± 0.22^c^
GSH-Px (U/mg prot)	502.29 ± 24.34^a^	863.55 ± 86.64^b^	945.35 ± 50.86^bc^	1059.39 ± 31.97^c^

Different lowercase letters in the same trade indicate significant differences (*P* < 0.05).

MDA, malondialdehyde; T-AOC, total antioxidant capacity; CAT, catalase; SOD, superoxide dismutase; GSH-Px, glutathione peroxidase.

### 3.3 Plasma components

**Table 5** illustrates that dietary *C. butyricum* influenced the plasma composition of hybrid grouper. Compared to the CON group, the C4 content and LZM activity were significantly elevated in the other groups (*P* < 0.05). Furthermore, the activities of AST and ALT were unaffected by the dietary treatments (*P* > 0.05).

**Table 5:** Effects of dietary *C. butyricum* addition on plasma components of hybrid grouper.

Parameters	Diets			
	CON	CB1	CB2	CB3
AST (U/L)	54.15 ± 0.96	54.47 ± 2.63	50.54 ± 0.31	54.23 ± 4.67
ALT (U/L)	83.98 ± 2.36	86.24 ± 0.56	82.28 ± 0.22	77.98 ± 4.17
IgM (μg/ml)	414.95 ± 22.96^a^	513.65 ± 24.66^bc^	485.26 ± 0.81^b^	575.58 ± 8.46^c^
C4 (μg/ml)	153.54 ± 28.28^a^	229.93 ± 9.91^bc^	202.02 ± 2.07^b^	265.12 ± 32.57^c^
LZM (U/ml)	112.40 ± 1.03^a^	145.46 ± 30.67^b^	149.56 ± 1.34^b^	137.87 ± 28.48^b^

Different lowercase letters in the same trade indicate significant differences (*P* < 0.05).

AST, aspartate aminotransferase; C4, complement 4; ALT, alanine aminotransferase; IgM, immunoglobulin M; LZM, lysozyme.

### 3.4 Intestinal histomorphology

**Figure 1:**
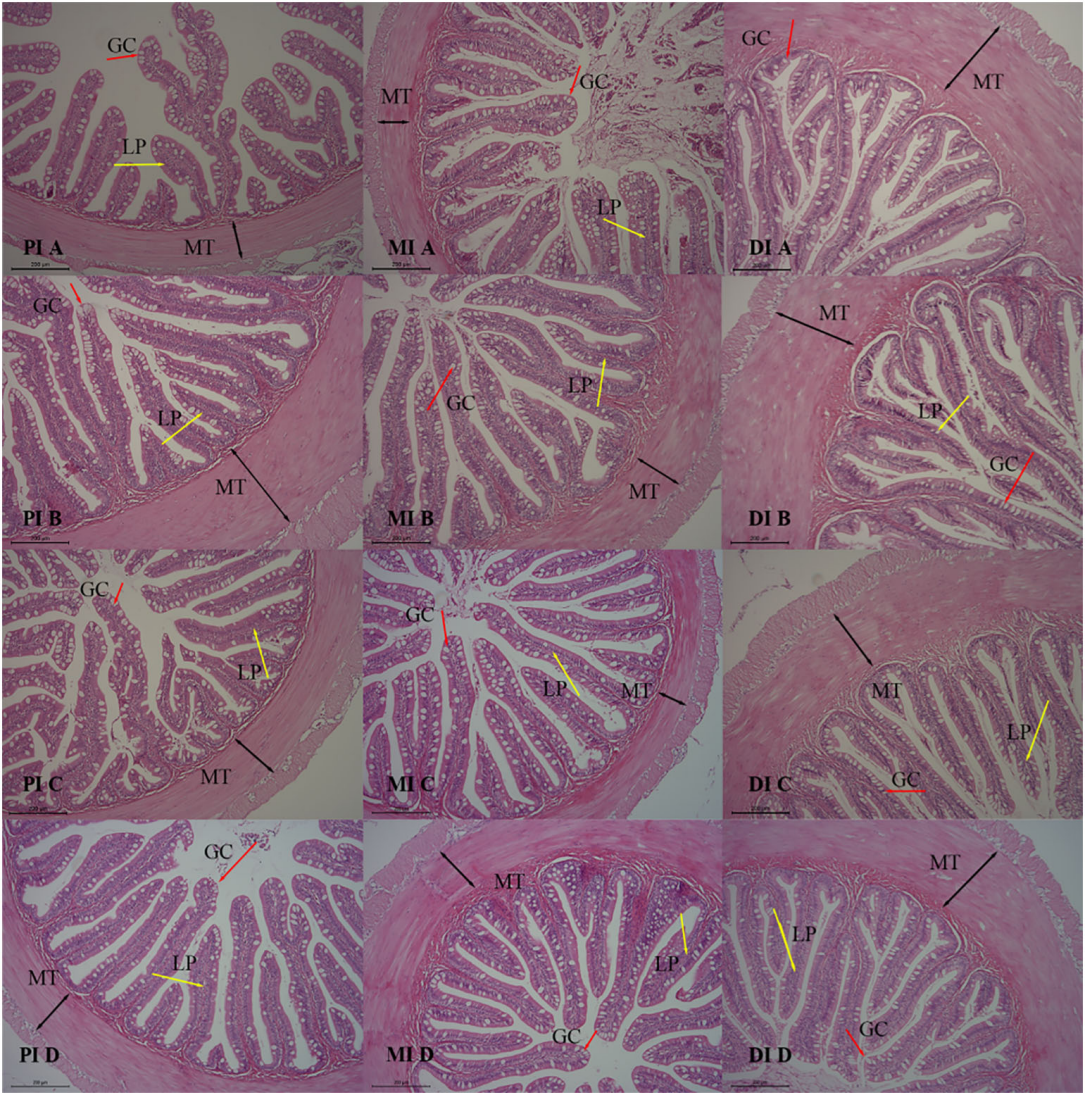
Effects of dietary *C. butyricum* addition on the intestinal morphology of hybrid grouper (100×). A, B, C and D represent the CON group, CB1 group, CB2 group and CB3 group, respectively. Muscle thickness (MT) is indicated by black bidirectional arrows, the lamina propria (LP) by yellow unidirectional arrows, and goblet cells (GC) by red unidirectional arrows.

As shown in **Table 6**, the muscle layer thickness (MT) and mucosal fold height (HMF) of the midgut were significantly increased in the *C. butyricum* group compared to the CON group (*P* < 0.05). The inclusion of *C. butyricum* in the feed had no effect on the morphological indicators of the anterior and posterior intestinal tissues of hybrid grouper (*P* > 0.05). The observations of intestinal tissue slice are presented in **Figure 1**. As shown in **Figure 1**, the tissue structure of the anterior and middle intestines in the CB1, CB2, and CB3 groups appears clearer and more structurally intact than that of the CON group, with well-developed mucosal folds. The results indicated that adding *C. butyricum* to the feed significantly improved the tissue structure of the anterior and middle intestines of hybrid grouper, with the CB2 group demonstrating the most pronounced improvements.

**Table 6:** Effects of dietary *C. butyricum* addition on intestinal histomorphology of hybrid grouper.

Parameters	Diets			
	CON	CB1	CB2	CB3
Proximal intestine				
MT (μm)	189.82 ± 43.87	206.65 ± 58.56	169.01 ± 23.76	190.96 ± 13.37
NFM (unit)	61.50 ± 6.50	57.00 ± 6.00	61.5 ± 3.5	47.50 ± 1.50
HMF (μm)	459.73 ± 36.73	447.51 ± 5.72	440.92 ± 53.13	422.9 ± 48.26
Middle intestine				
MT (μm)	104.06 ± 12.43^a^	134.57 ± 20.7b	132.84 ± 1.85^b^	155.48 ± 13.82c
NFM (unit)	33.50 ± 0.50^a^	40.50 ± 0.50b	34 ± 4.0^ab^	31.50 ± 2.50a
HMF (μm)	351.19 ± 29.99a	385.85 ± 15.60ab	402.33 ± 1.59^b^	392.77 ± 8.28ab
Distal intestine				
MT (μm)	224.40 ± 94.44	201.77 ± 35.61	190.31 ± 53.1	238.04 ± 2.29
NFM (unit)	51.50 ± 6.50	51.00 ± 3.00	58.5 ± 0.5	45.50 ± 2.50
HMF (μm)	465.42 ± 90.64	382.82 ± 51.13	432.03 ± 15.07	376.88 ± 36.84

Different lowercase letters in the same trade indicate significant differences (*P* < 0.05).

MT, muscle layer thickness; NFM, mucosal fold number; HFM, mucosal fold height.

### 3.5 Microbiological analysis

**Figure 2:**
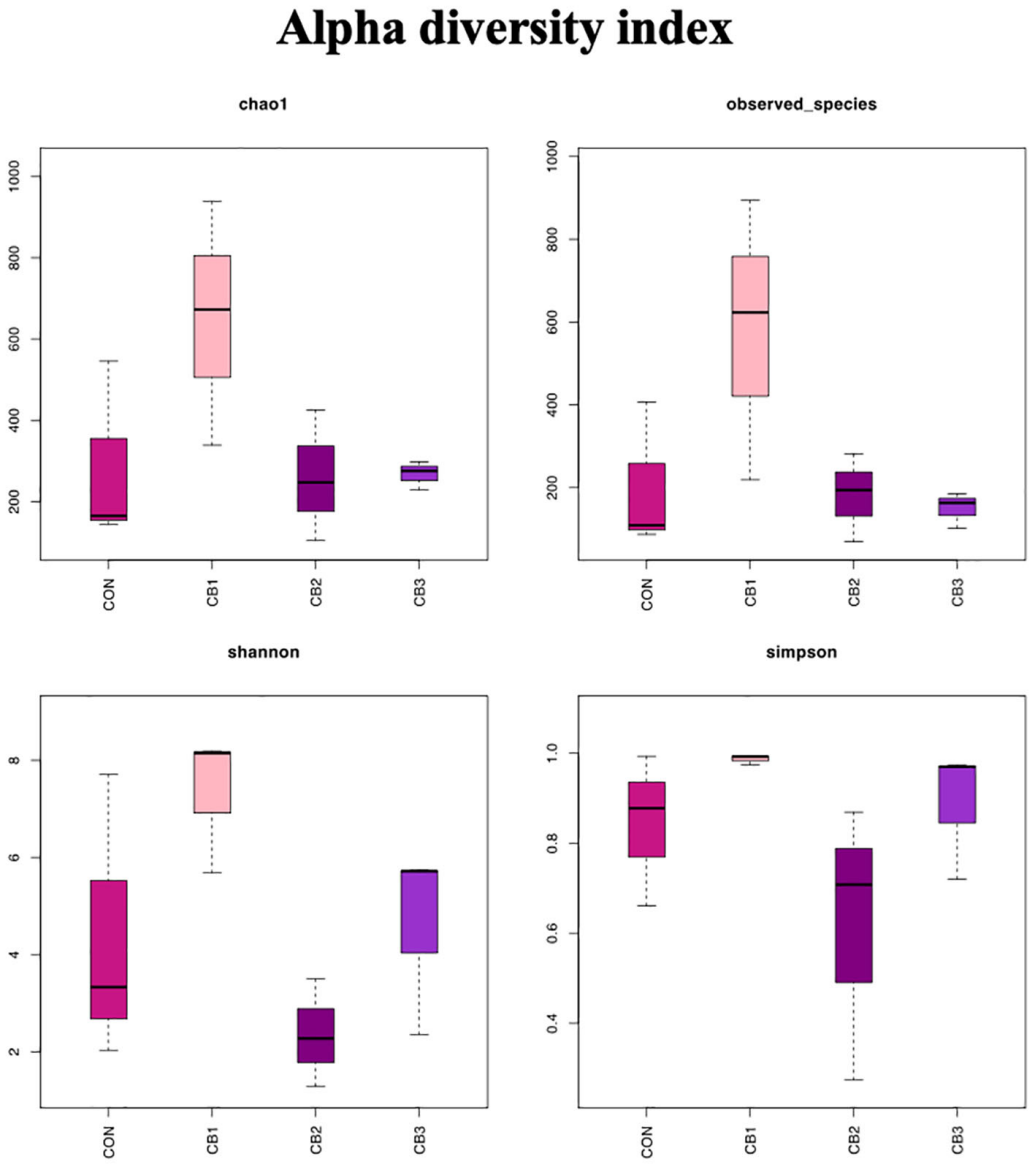
Alpha diversity indices of the gut microbiota in hybrid grouper.

By comparing the alpha diversity indices of microbial communities in different treatment groups, significant differences in species richness and diversity were observed among the groups (**Figure 2**).

Chao1 index: The CB1 group exhibited the highest estimated species richness, followed by the CON group, while the CB2 and CB3 groups exhibited significantly lower species richness.

Observed species index: The CB1 group had the highest number of observed species, followed by the CON group, while the CB2 and CB3 groups had lower numbers of observed species.

Shannon index: The Shannon diversity index of the CB1 group was the highest, indicating the highest community diversity and evenness. The CON group ranked second, while the CB2 and CB3 groups exhibited lower levels.

Simpson index: The CB1 group had the highest Simpson diversity index, further demonstrating that the CB1 group exhibited the highest community diversity and stability. The CON group ranked second, while the CB2 and CB3 groups exhibited lower levels.

**Figure 3:**
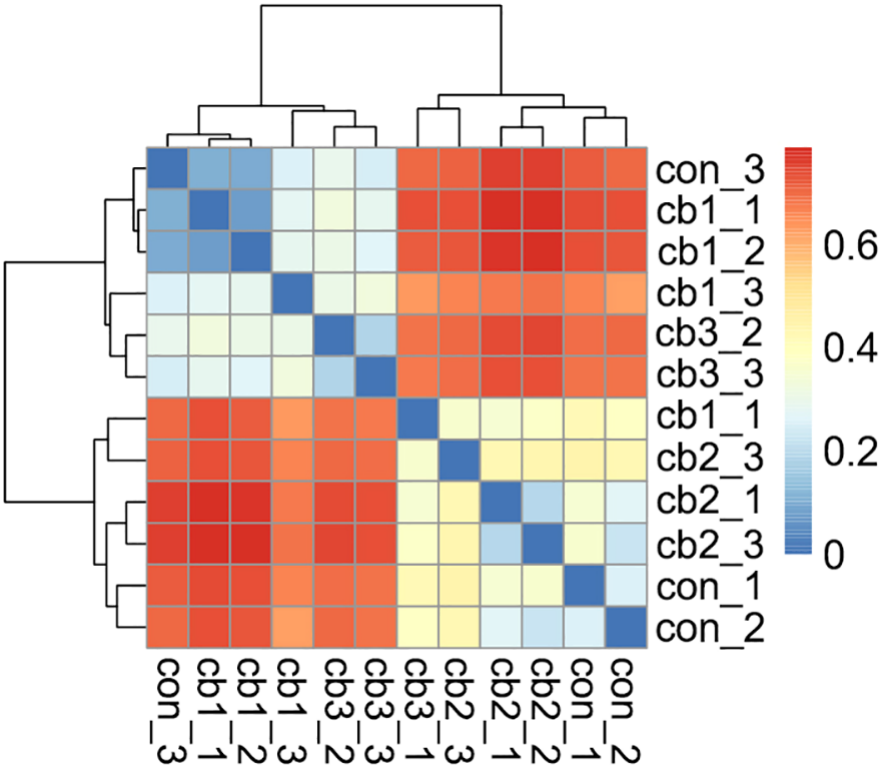
Beta diversity indices – UniFrac heatmap of the gut microbiota in hybrid grouper.

Based on the heatmap and hierarchical clustering tree, β-diversity analysis revealed significant differences in microbial community structure among the treatment groups (**Figure 3**). Samples within the same group (e.g., con_1, con_2, con_3 in the CON group) exhibited a predominance of blue coloration in the heatmap, indicating high similarity among these samples. In contrast, samples from different groups (e.g., the CON group compared with the CB1 group) displayed a predominance of red coloration in the heatmap, indicating lower similarity between these groups. The hierarchical clustering tree illustrated the relationships between samples, with highly similar samples clustering together. The results indicate that samples within the same group shared high similarity, while significant intergroup differences were observed. Specifically, the CON group exhibited low similarity with the CB1 and CB3 groups, while a higher similarity was observed with the CB2 group. These findings highlight the significant structural variations in microbial communities among the different treatment groups (CON, CB1, CB2, and CB3).

**Figure 4:**
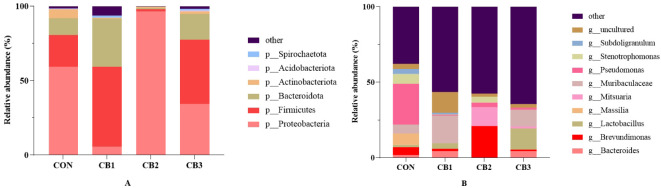
Classification diagram of intestinal microbiota in each group at the phylum **(A)** and genus **(B)** level of the hybrid grouper.

The classification diagram of intestinal microorganisms at the phylum level among groups is shown in **Figure 4A** and **Supplementary Table 1**. At the phylum level, the dominant microflora in the intestine of hybrid grouper were Proteobacteria, Firmicutes, and Bacteroidota, accounting for more than 90%. A higher relative abundance of Firmicutes and Bacteroidota and a lower relative abundance of Proteobacteria were observed in the CB1 and CB3 groups compared to the CON group, and CB2 exhibited the opposite trend. In addition, although not significantly different among groups (*P* > 0.05), the relative abundance of Actinobacteriota was lower in the CON group than in any of the experimental groups.

The classification diagram of intestinal microorganisms at the genus level among groups is shown in **Figure 4B** and **Supplementary Table 1**. At the genus level, the bacterial communities in the dietary treatments included the following genera in particular: *Bacteroides*, *Brevundimonas*, *Lactobacillus*, *Mitsuaria*, *Massilia*, *Pseudomonas*, *Stenotrophomonas* and *Subdoligranulum*. Decreased abundances of the genera *Massilia*, *Pseudomonas*, *Stenotrophomonas*, and *Subdoligranulum*, and increased abundances of the genera *Lactobacillus*, *Bacteroides* and *Mitsuaria* were observed in the experimental groups compared to the CON group.

### 3.6 Expression of tight junction structural protein and intestinal inflammatory factor genes

**Figure 5:**
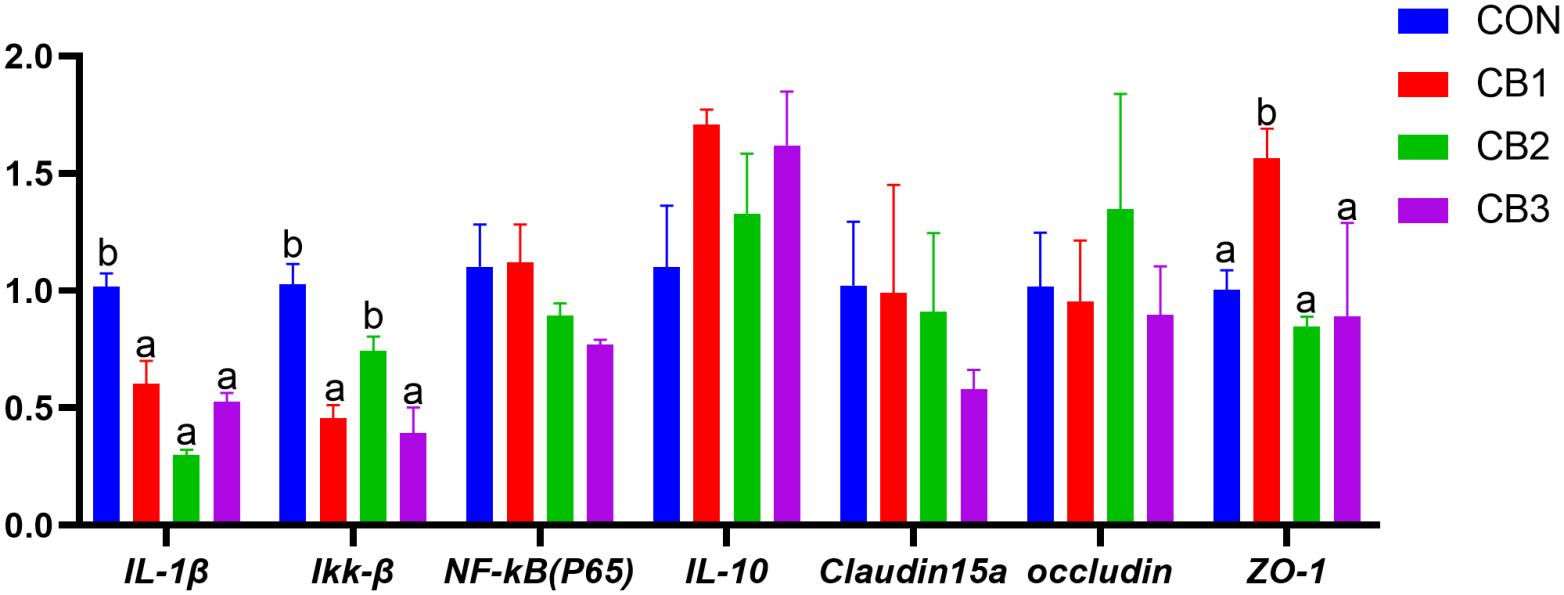
Effects of dietary *C. butyricum* on the gene expression levels of intestinal inflammatory factors and tight junction structural protein in hybrid grouper. Different lowercase letters in the same trade indicate significant differences (*P* < 0.05).

The effect of dietary *C. butyricum* supplementation on the expression levels of intestinal inflammatory factors and tight junction structural protein genes in hybrid grouper were illustrated in **Figure 5**. Compared to the CON group, the gene expression levels of pro-inflammatory factors *IL-1β* and *Ikk-β* in the experimental groups generally exhibited a down-regulation trend (*P* < 0.05), whereas the gene expression level of anti-inflammatory factor *IL-10* was up-regulated, although no significant differences were observed (*P* > 0.05). Regarding tight junction protein genes, the expression levels of the *ZO-1* gene in the CB1 group were significantly higher than those in the CON group (*P* < 0.05). However, no significant differences were found in the expression levels of claudin 15a and occludin proteins (*P* > 0.05).

## 4 Discussion

In this study, the addition of *C. butyricum* strains from different sources to the diets had no discernible effect on the growth performance of hybrid grouper. This outcome is consistent with the observations reported for other animals, including carp (45, 46), Chinese drum (47), and goats (48). Incorporating *C. butyricum* into a dietary formulation comprising a low fish meal content has been shown to significantly promote fish growth (17, 25, 49). This effect may be attributed to *C. butyricum*’s ability to enhance intestinal metabolic function and facilitate nutrient digestion and absorption through the secretion of a multitude of short-chain fatty acids and digestive enzymes within the intestine (45, 50). These disparities may be due to the fact that the protein and fat levels in the diets are tailored to meet the nutritional requirements of hybrid grouper in this study. Consequently, the incorporation of *C. butyricum* did not result in a pronounced growth-promoting effect in hybrid grouper, suggesting that the growth-promoting effects of *C. butyricum* cannot be fully realized by merely incorporating it into feeds with high-quality protein sources.

The antioxidant capacity of the body serves as an indicator of overall health and well-being. During physiological stress or illness, the body generates an excess of free radicals, leading to oxidative damage (51). The intestine, as a conduit between the internal and external environments, is constantly exposed to free radicals from both exogenous substances and endogenous metabolites, resulting in intestinal oxidative damage (52). Previous studies have demonstrated that administering *C. butyricum* to animals enhances antioxidant enzyme activity and reduces MDA levels (17, 28, 33, 53, 54), thus preventing damage to the body, which is consistent with the present study’s findings. The results of the present study demonstrated that dietary *C. butyricum* administration led to a significant increase in SOD and GSH-Px levels in the intestine of hybrid grouper, along with a decrease in MDA levels. The antioxidant effect of *C. butyricum* is linked to its production of short-chain fatty acids, such as butyric and propionic acids, in the intestinal lumen, providing an energy source for intestinal mucosal cells and supporting intestinal homeostasis (55). Additionally, *C. butyricum* can produce nicotinamide adenine dinucleotide phosphate, scavenge reactive oxygen species, and enhance antioxidant potential, which may further contribute to the observed improvement in antioxidant capacity (56).

The present study demonstrated that dietary *C. butyricum* administration led to a significant increase in serum LZM levels, with the CB1 group showing a marked elevation in serum IgM. These findings indicate that dietary supplementation with *C. butyricum* enhances immune competence in fish. The observed outcomes were consistent across diverse species, including *Miichthys miiuy* (57) silver pomfret (24) large yellow croaker (58), tilapia (17), shrimp (53), and gibel carp (30). These results may be attributed to increased IgM and LZM levels, which are known to enhance the organism’s capacity to eliminate pathogens and pathogenic microorganisms (59, 60). Furthermore, the incorporation of *C. butyricum* into the diet elevated the serum C4 concentration of tilapia (11) and broilers (61, 62), thereby reinforcing the immune system, findings corroborated by this study.

The integrity of the intestinal tissue structure in fish is essential for the efficient maintenance of nutrient digestion and absorption (63). Greater intestinal plica height corresponds to an increased intestinal surface area and improved nutrient absorption capacity (64). Additionally, intestinal muscular layer thickness reflects its peristaltic capacity, which affects chyme digestion rate and intestinal emptying (65). Short-chain fatty acids act as an energy source for epithelial cells, promoting the growth and development of intestinal villi, while mitigating mucosal injury and reducing intestinal cell apoptosis (33). Our experimental findings, consistent with prior studies (17, 27), demonstrate that the addition of *C. butyricum* to the feed effectively improves the intestinal structure in fish.

Furthermore, *C. butyricum* enhances intestinal health by modulating the intestinal microbiota. This study identifies Proteobacteria, Firmicutes, and Bacteroidetes as the dominant intestinal flora in hybrid grouper, consistent with previous studies on grouper (66). The addition of *C. butyricum* significantly altered the dominant intestinal flora, increasing the relative abundance of Firmicutes and Bacteroidetes while reducing that of Proteobacteria and Actinobacteria. This finding is consistent with studies on *Epinephelus coioides* (67) and *Procambarus clarkii* (68). Firmicutes and Bacteroidetes are known to produce substantial amounts of short-chain fatty acids (69, 70), which may partly explain why *C. butyricum* increases short-chain fatty acid content in the intestine. Many bacteria within Proteobacteria and Actinobacteria are considered potential pathogens, and their increased relative abundance may disrupt microbial balance, increasing the risk of enteritis or pathogen invasion (71, 72). In this study, *C. butyricum* significantly decreased the relative abundance of these two phyla, suggesting its potential to mitigate the risk of enteritis by improving microbial structure and enhancing intestinal health.

This study evaluated the impact of *C. butyricum* supplementation on the expression of intestinal inflammatory cytokine and tight junction (TJ) protein genes in hybrid grouper (*E. lanceolatus × E. fuscoguttatus*). The results demonstrated that *C. butyricum* supplementation significantly regulated inflammation and improved intestinal barrier integrity. Pro-inflammatory cytokine gene expression (e.g., *IL-1β* and *Ikk-β*) was significantly downregulated in the experimental group, consistent with findings reported by Lu et al. (73). These cytokines are critical mediators of the inflammatory response, and their overexpression is closely linked to tissue damage and compromised intestinal function (74).

Beyond its anti-inflammatory effects, *C. butyricum* supplementation significantly upregulated ZO-1 expression levels. Tight junctions (TJs) are critical intercellular connections. Occludin, a key functional protein, interacts with tight junction proteins such as ZOs, playing an indispensable role in biological barrier function and tight junction formation. ZO proteins, particularly ZO-1, link occludin to the actin cytoskeleton and play a vital role in maintaining tight junction integrity, reducing the risk of intestinal tumors and related disease (75, 76). This study revealed that *C. butyricum* supplementation significantly upregulated ZO-1 expression levels. This crucial TJ protein enhances intestinal barrier integrity and elasticity by regulating paracellular permeability, potentially reducing the risk of pathogen and endotoxin translocation into systemic circulation (77). Notably, no significant changes were observed in claudin-15a and occludin expression levels, indicating that *C. butyricum* may selectively regulate specific TJ proteins.

## 5 Conclusions

In this study, the addition of *C. butyricum* to the diet did not significant impact on the growth performance of hybrid grouper but notably enhanced intestinal antioxidant enzyme activity and improved intestinal morphology and structure. From the perspective of intestinal flora composition, tight junction proteins, and inflammatory gene expression, the addition of *C. butyricum* to the diet reduced intestinal inflammation, promoted the proliferation of beneficial bacteria, and suppress**ed** pathogenic bacteria. These findings highlighted its potential role in promoting intestinal structural integrity and health.

## Data Availability

The datasets presented in this study can be found in online repositories. The names of the repository/repositories and accession number(s) can be found below: https://www.ncbi.nlm.nih.gov/genbank/, KF861990.1 https://www.ncbi.nlm.nih.gov/genbank/, MK809394 https://www.ncbi.nlm.nih.gov/genbank/, MK809396 https://www.ncbi.nlm.nih.gov/genbank/, EF582837.1 https://www.ncbi.nlm.nih.gov/genbank/, KM669150.1 https://www.ncbi.nlm.nih.gov/genbank/, EU219847.1 https://www.ncbi.nlm.nih.gov/genbank/, EF582837.1.
