# Supplementary material for: Effect of dietary Clostridium butyricum supplementation on growth performance, immune function, and intestinal health of hybrid grouper (Epinephelus fuscoguttatus ♀ × Epinephelus lanceolatus ♂)
Source: Front Immunol. 2025 Feb 26;16:1557256. doi: 10.3389/fimmu.2025.1557256 (PMC11897522; doi:10.3389/fimmu.2025.1557256)
Supplement: Supplementary file 1 [file Table1.pdf]

Supplementary material

Table 1. Effects of dietary *C. butyricum* addition on the classification diagram of intestinal microbiota in each group at the phylum and genus level of hybrid grouper (%)

Parameters	Diets			
	CON	CB1	CB2	CB3
		phylum		
Acidobacteriota	0.11±0.11	0.23±0.12	0.09±0.09	0.01±0.01
Actinobacteriota	6.16±5.72	0.44±0.18	0.84±0.76	0.77±0.41
Bacteroidota	11.22±11.19[ab]	32.47±3.46[b]	0.88±0.56[a]	12.16±6.68[ab]
Firmicutes	21.22±16.37	53.54±1.28	1.28±0.78	27.18±18.49
Proteobacteria	59.39±26.88	5.70±2.62	96.53±1.52	57.22±25.11
Spirochaetota	0.31±0.31	1.26±0.63	0.00±0.00	0.56±0.56
other	1.58±1.57	6.37±0.68	0.39±0.19	2.11±1.12
		genus		
Bacteroides	1.65±1.64	4.23±0.58	0.24±0.10	2.30±2.28
Brevundimonas	5.26±4.93	1.65±1.54	20.67±13.78	12.92±12.86
Lactobacillus	1.68±1.42[a]	3.61±0.97[a]	0.03±0.03[a]	11.05±5.54[ab]
Massilia	7.36±7.36	0.03±0.02	0.03±0.03	0.02±0.01
Mitsuaria	0.03±0.03	0.04±0.04	12.38±6.80	13.96±13.95
Muribaculaceae	6.06±6.06	18.43±1.34	0.09±0.09	4.97±4.97
Pseudomonas	26.77±16.84	0.49±0.40	3.12±0.88	17.58±9.62
Stenotrophomonas	6.69±6.69	0.31±0.31	3.71±3.68	0.01±0.00
Subdoligranulum	3.17±2.88	0.84±0.30	0.05±0.04	0.00±0.00
uncultured	3.44±3.43[a]	13.72±0.67[b]	2.13±1.54[a]	3.17±2.05[a]
other	37.88±11.52	56.65±0.67	57.54±19.50	34.03±10.66

Different lowercase letters in the same trade indicate significant differences ($P < 0.05$)
